# Adsorption Performance of a Multimodal Anion-Exchange Chromatography Membrane: Effect of Liquid Phase Composition and Separation Mode

**DOI:** 10.3390/membranes12121173

**Published:** 2022-11-22

**Authors:** Tomáš Kurák, Milan Polakovič

**Affiliations:** Department of Chemical and Biochemical Engineering, Faculty of Chemical and Food Technology, Institute of Chemical and Environmental Engineering, Slovak University of Technology, Radlinského 9, 812 37 Bratislava, Slovakia

**Keywords:** membrane chromatography, multimodal adsorption, BSA/DNA separation, binding capacity, desorption recovery, flow-through mode, bind-and-elute mode, membrane reuse

## Abstract

Membrane chromatography is a modern, high-throughput separation method that finds important applications in therapeutic protein purification. Multimodal, salt-tolerant membranes are the most recent innovation in chromatographic membrane adsorbents. Due to the complex structure of their ligands and the bimodal texture of their carriers, their adsorption properties have not been sufficiently investigated. This work deals with the equilibrium and kinetic properties of a multimodal anion-exchange chromatography membrane, Sartobind STIC. Single- and two-component adsorption experiments were carried out with bovine serum albumin (BSA) and salmon DNA as model target and impurity components. The effect of the Hofmeister series ions and ionic strength on the BSA/DNA adsorption was investigated in micromembrane flow experiments. A significant difference was observed between the effects of monovalent and polyvalent ions when strong kosmotropic salts with polyvalent anions acted as strong displacers of BSA. On the contrary, DNA binding was rather high at elevated ionic strength, independent of the salt type. Two-component micromembrane experiments confirmed very high selectivity of DNA binding at a rather low sodium sulfate feed content and at pH 8. The strength of binding was examined in more than a dozen different desorption experiments. While BSA was desorbed relatively easily using high salt concentrations independent of buffer type and pH, while DNA was desorbed only in a very limited measure under any conditions. Separation experiments in a laboratory membrane module were carried out for the feed containing 1 g/L of BSA, 0.3 g/L of DNA, and 0.15 M of sodium sulfate. The negative flow-through mode was found to be more advantageous than the bind-elute mode, as BSA was obtained with 99% purity and a 97% yield. Membrane reuse was investigated in three adsorption-desorption-regeneration cycles.

## 1. Introduction

Membrane chromatography is a relatively new purification technique in the bioseparation field. Membrane chromatographic adsorbents were developed based on the principles of filtration membranes. Advantages of membrane filters include easy scaling, disposability, and often rapid and robust performance in a single pass. The same advantages apply to membrane adsorbers.

The most attractive feature of membrane chromatography is its high throughput. As solute transport is largely based on convection, the time needed for a solute to reach its binding site during adsorption and away from it during elution is significantly lower than in traditionally column chromatography using resin beads, where transport is controlled by the intra-particle diffusion [1,2]. The throughput of membrane chromatography is therefore faster, which contributes to a higher productivity and a decrease in product degradation by proteolysis, denaturation, and aggregation [3,4]. Another advantage of adsorptive membranes is that their binding capacity, expressed through the mass of bound biopolymer, increases with its molecular weight [5,6]. On the contrary, the binding capacity of adsorptive beads decreases since many impurities of mega-Dalton size cannot effectively access the pore structure.

Membrane chromatography is thus a useful large-scale separation process for the purification and recovery of nucleic acids and large proteins, as well as for the removal of trace impurities or potential contaminants such as plasmid DNA, endotoxins, and viruses [7]. Moreover, the disposable nature of the membrane chromatography devices eliminates the need for equipment cleaning and revalidation, thereby accelerating downstream processes in order to keep pace with upstream productivity in the production of monoclonal antibodies [8,9].

Membrane ligand chemistries cover the whole spectrum of applications, from ion-exchange [10,11,12] through hydrophobic interaction [7] to affinity chromatography [13,14]. Many downstream processes consist of at least two or more chromatographic steps to produce pure biomolecules. Downstream chromatographic steps are usually classified as capture, purification, and polishing. One of the most common chemistries used in the purification step is the quaternary amine (Q) chemistry of anion exchangers (AEX). AEX membrane adsorbers are mostly operated in the so-called negative flow-through mode, where impurities are bound and the product is allowed to pass through the membrane [15,16].

AEX membrane adsorbers with Q chemistry provide good performance only at low feed conductivity. Grafted hydrogel layers on a macroporous support collapses at high salt concentrations and can no longer be accessed by macromolecules and viruses [17]. This limitation often requires the dilution of the outlet stream from the preceding step or the addition of a diafiltration step. Feed dilution results in increased volumes for mixers, holding tanks, and buffers, along with added processing time [15]. In response, novel multi-mode membrane adsorbents with increased binding capacities at high salt concentrations have been developed [18]. Ligands of mixed-mode materials typically provide a combination of multiple binding modes such as ion exchange, hydrogen bonding, and hydrophobic interactions [19]. The primary mode of interaction between biological molecules and oppositely charged, immobilized ligands is electrostatic interaction. Additional interactions such as hydrophobic, van der Waals, and hydrogen bonding may also have a significant impact on the selectivity and allow for improved adsorption in solutions with high salt concentrations [20]. For the successful application of such an adsorbent, it is crucial to know the conditions under which a certain interaction is dominant and under which it disappears. To explore the full potential of multimodal adsorbents, the optimization of process conditions is essential.

**Figure 1:**
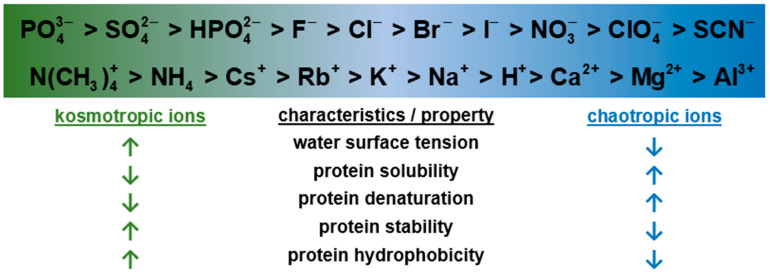
Hofmeister series of anions and cations and their influence on protein solution properties.

The relative importance of these interactions is determined by biomolecule properties, mobile phase, and resin chemistry [21]. Different mobile phase compositions can modulate the interactions of the target molecule with multimodal chromatography media. pH affects not only the net charge of proteins but also their solubility and hydrophobicity. [22,23]. Salts show two types of effects, nonspecific and specific [24]. Nonspecific salt effects are simply due to their ionic properties, are independent of the type of salt, and play a major role in ion exchange chromatography (IEX). Protein binding and elution are based on either charge shielding or stoichiometric ion binding and can be modulated by ionic strength. On the other hand, specific effects are dependent on the salt type and are often explained through Hofmeister series ions, as shown in Figure 1 [25]. They play an enormous role in hydrophobic interaction chromatography, where a kosmotropic salt has to be added to promote binding. The salt-specific effects are however important also in multimodal chromatography due to the above-mentioned significance of hydrophobic interactions [26,27]. The effect of salt type was investigated for the binding of lysozyme on a multimodal cation exchanger up to the salt concentration of 3 M [26]. Although there were differences among the four technically relevant salts (sodium chloride, sodium sulfate, ammonium chloride, and ammonium sulfate), they were relatively minor compared to the effects of pH and salt concentration. The effect of salt type was also investigated for a multimodal anion-exchange membrane [27]. Salt forms of acetate, citrate, chloride, and phosphate were investigated for the binding of BSA in the limited concentration range up to 125 mM. Citrate and phosphate ions suppressed BSA binding significantly. On the contrary, acetate and chloride ions slightly enhanced BSA binding. However, no positive effect was observed for human IgG. The choice of salt type thus plays an important role.

In this work, the influence of chromatographic conditions on the adsorption of bovine serum albumin (BSA) and salmon DNA on the multimodal salt-tolerant anion-exchange membrane is examined. These two compounds are commonly used as representative separation mixture component targets [27]. Sartobind STIC based on a regenerated cellulose support with a poly(allylamine) ligand [28] was investigated. For this purpose, a micromembrane chromatography module based on a 96-well plate was used for screening to determine optimum adsorption conditions in terms of pH, salt type, and conductivity. First, the influence of pH and buffer type was examined. Then, the dependence of adsorbed amount on the salt type and of ionic strength on the binding capacity were investigated for a wide range of anions and cations from the Hofmeister series. The influence of eluent composition and the time interval between the adsorption and desorption phases on the recovery of adsorbed molecules was also investigated. Finally, dynamic experiments were performed for the selected conditions in both negative flow-through and bind-and-elute modes. The reuse of the membrane was also examined for the negative flow-through mode.

## 2. Experimental Methods

### 2.1. Adsorbents

Salt-tolerant interaction chromatography membrane, Sartobind STIC, was kindly donated by Sartorius Stedim Biotech (Göttingen, Germany). Sartobind STIC was either in the form of A4 sheets or built-in micromembrane modules [29]. The membranes shipped by the producer were impregnated with glycerol which was washed out with sufficient volumes of redistilled water before each experiment.

### 2.2. Chemicals

Salts (NH_4_Cl, NaCl, (NH_4_)_2_SO_4_, Na_2_SO_4_, MgCl_2_ 6H_2_O, NaF, and K_2_HPO_4_) and chemicals for buffer preparation (tris, bis-tris, bis-tris propane, and phosphate) were from Centralchem (Banská Bystrica, Slovakia). GuHCl, NaSCN, and bovine serum albumin (A4503-50G) were purchased from Merck (Darmstadt, Germany). Salmon sperm DNA (A2159) was obtained from PanReac AppliChem ITW Reagents (Darmstadt, Germany).

### 2.3. Membrane Characterization

Membrane thickness was determined using a contact thickness gauge from MTS Systems (Cary, NY, USA) and it was 0.275 mm. To obtain quantitative information about this membrane’s porous structure, the same characterization methods were applied as for an ion-exchange membrane with a grafted gel layer in our previous work [17]. The specific total pore volume and total porosity were determined by a liquid impregnation method. The amount (volume) of the water filling the pore space of the membrane was determined by an infrared moisture analyzer, the KERN MLS-C (KERN & SOHN GmbH, Balingen, Germany).

The specific pore volume of transport pores was determined by batch size exclusion using 2 M dextran [17]. The molecules of 2 M dextran can be considered as completely excluded from adsorptive pores. This method is based on the submerging of a predefined amount of a membrane sample into a solution of a particular solute at a known concentration for a certain period of time. Subsequently, the decrease in the solute concentration due to the size-exclusion effect was measured. The equilibrium concentration of 2 M dextran was determined by measuring with an Agilent 1100 Series refractive index detector (Agilent Technologies, Santa Clara, CA, USA). The specific pore volume was calculated using the following equation:

(1) $$v_{d}=\left(\frac{c_{0}}{c_{\mathrm{eq}}}-1\right)\frac{V_{s}}{m_{m}}$$

where $c_{0}$ and $c_{\mathrm{eq}}$ are the dextran initial and equilibrium concentrations, respectively. $V_{s}$ is the volume of solution in contact with a membrane sample and $m_{m}$ is the mass of the dry membrane.

The inverse size-exclusion chromatography method was used to determine the pore size distribution of secondary pores [17]. Experiments were conducted employing a Tricorn 5/100 column (GE Healthcare Bio-Sciences AB, Uppsala, Sweden) packed with membrane stripes (2.5 × 50 mm) to form a compact bed with a length of 5 cm. Solute probes with different molecular sizes (glucose and dextrans (Fluka BioChemika, Buchs, Switzerland) with molecular weights from 1200 g/mol to 2,000,000 g/mol) were injected into the column. The column outlet concentration was monitored by means of refractive index (RI) detection. The pore distribution (partition) coefficients were evaluated using the solute retention times determined by the moment method.

(2) $$D_{p}=\frac{t_{i}-t_{\max}}{t_{\min}-t_{\max}}$$

where $t_{i}$ is retention time of injected solute probe, $t_{\max}$ and $t_{\min}$ are retention times of the largest dextran and glucose, respectively.

### 2.4. Micromembrane Adsorption

#### 2.4.1. Centrifuge-Forced Flow

Micromembrane modules kindly donated by Sartorius Stedim Biotech were used to investigate the influence of mobile phase composition (buffer type, pH, and salts) on BSA and DNA single-component adsorption. The upper part of the micromembrane module is a Vivawell-96-well plate with 96 fixed open wells with a volume of 600 μL. The bottom of each well is formed by three membrane layers with a total volume of 19 μL. The lower part of the module is a collection plate containing wells for the solution flowing through the micromembranes.

Flow through the micromembranes was achieved by centrifugation at a rotation frequency of 500 rpm for 2 min and subsequently at a rotation frequency of 2000 rpm for 1 min. These centrifugation conditions were tested and found to be sufficient to transfer the whole loaded volume through the micromembrane.

Micromembranes were first flushed three times with 500 μL of redistilled water. They were then equilibrated twice with 500 μL of a 20 mM buffer. The loading phase was carried out by stepwise feeding of 500 μL of a buffered solution containing either 1 g/L of BSA or 0.3 g/L of DNA. For the determination of adsorption isotherms, a binding solution containing 0.05–2.5 g/L BSA or 0.015–0.6 g/L DNA in 20 mM tris buffer with pH 8 was used in the loading phase. The collection plate containing the flow-through fraction was transferred into an automatic injection sampler of the HPLC workstation Agilent 1100 (Santa Clara, CA, USA), which was used to determine the BSA or DNA concentration from the UV absorbance at 280 nm or 260 nm, respectively. If the flow-through concentration was not very close to the feed concentration, the loading phase continued with another feeding step [29]. BSA and DNA-specific adsorbed amounts, $q_{BSA}$ and $q_{DNA}$, respectively, were obtained from the following equation:

(3) $$q_{i}=\frac{V_{L}\left(c_{Li}-c_{i}\right)}{V_{m}}$$

where subscript i represents either BSA or DNA, $c_{Li}$ the feed concentration, $c_{i}$ the flow–through concentration (calculated as the mean of all loading steps), $V_{L}$ the total feed volume, and $V_{m}$ the membrane volume.

Adsorption/desorption experiments were conducted so that the microplate was first loaded with 2 mL of loading solution containing 1 g/L BSA or 0.3 g/L DNA, both in 20 mM tris buffer with pH 8. After the loading phase, the microplates were washed with 1.5 mL of loading buffer. The desorption phase with 2 mL of elution solution (different eluents) took place immediately after the washing step or was postponed for 12 h, during which the membrane with adsorbed BSA/DNA was incubated in the loading buffer. At each step, the amounts of adsorbed and desorbed protein were determined from the mass balance. Recovery of adsorbed BSA/DNA was calculated using the following equation:

(4) $$\mathrm{Recovery}=\frac{V_{W}c_{Wi}+V_{E}c_{Ei}}{V_{L}\left(c_{Li}-c_{i}\right)}100\%$$

where $V_{W}$ and $V_{E}$ are the solution volumes used in the washing and elution steps, respectively, and $c_{Wi}$ and $c_{Ei}$ are the concentrations in the flow-through after the wash and elution phases, respectively. All experiments using micromembrane modules were performed in triplicate.

#### 2.4.2. Pump-Forced Flow

In order to achieve continuous flow, a modified micromembrane module was constructed. A single well was cut from the Vivawell-96-well plate and fitted with special inlet and outlet adapters. The module was connected to the fast protein liquid chromatography (FPLC) system ÄKTA Purifier (GE Healthcare, Uppsala, Sweden).

This module was used in two-component adsorption experiments. Prior to the measurements, the micromembrane was washed with 3 mL of redistilled water and equilibrated with 3 mL of the equilibration buffer (20 mM tris buffer with pH 8). The feed was formed by 1 g/L of BSA and 0.3 g/L of a DNA mixture in 20 mM tris buffer with pH 8. It also contained different concentrations of either NaCl or Na_2_SO_4_. The feed flow rate was 0.5 mL/min, and its total volume was 2 mL. In order to monitor the breakthrough of non-adsorbed components, the outlet flow passed through a UV detector (260 nm). The outlet stream absorbance *A* was related to the feed absorbance *A*_0_.

The mean BSA concentration in flow–through was determined by the standard bicinchoninic acid assay (BCA). The flow–through samples were analyzed using an Agilent HPLC workstation. The UV signal obtained corresponded to the total BSA and DNA concentrations. The chromatogram was deconvoluted using the known mean BSA concentration, and the mean DNA concentration in flow–through was calculated. The adsorbed amounts of BSA and DNA were then obtained from Equation (3).

Equation (5) was used to calculate the distribution factors, $k_{\mathrm{BSA}}$ and $k_{\mathrm{DNA}}$,

(5) $$k_{i}=\frac{q_{i}}{c_{Li}}$$

The competition between BSA and DNA binding was quantified through selectivity factor, *S*,

(6) $$S=\frac{k_{\mathrm{DNA}}}{k_{\mathrm{BSA}}}$$

### 2.5. Laboratory Membrane Module Adsorption

Measurements were performed for a three-layer membrane configuration (with an effective membrane bed cross-sectional area of 5 cm^2^ and a total bed volume (BV) of about 400 µL) using a plastic membrane module with a flow distributor in a stainless steel holder connected to the low-pressure liquid chromatography system ÄKTA FPLC with a UV detector set to 260 nm [12]. The membrane module was provided by Sartorius Stedim Biotech (Göttingen, Germany).

In all experiments, the membrane module was first washed with 35 BV of redistilled water and equilibrated with 35 BV of loading buffer (20 mM tris buffer with pH 8). The effect of volumetric flow rate on the dynamic binding capacity of BSA was investigated for flow rates in the range of 1–70 mL/min. For these experiments, a solution with a BSA concentration of 1 g/L in the tris buffer with pH 8 was used as the feed solution.

The separation of BSA from DNA was conducted in both flow–through and bind-and-elute modes. In the flow–through mode, the feed contained 0.15 mol/L of Na_2_SO_4_, 1 g/L of BSA, and 0.3 g/L of DNA in the loading buffer. Loading was carried out using 70 BV of the loading solution. The bed was then washed with at least 20 BV of the loading buffer. The same buffer containing 2 M NaCl was used for elution. Regeneration was performed using 1 M NaOH. In the bind-and-elute mode, the feed was the same BSA/DNA mixture but did not contain Na_2_SO_4_. Before elution, the membrane was washed with 20 BV of buffer. Adsorbed components were eluted using 50 BV of 0.15 mol/L Na_2_SO_4_ in the buffer. All experiments were conducted at a flow rate of 5 mL/min. The dead volume of the chromatographic system was calculated from the residence time of a 2% acetone solution. Outlet fractions were collected during the loading, wash, and elution phases and then analyzed in the same manner as described in the previous section. The measured compositions were used to calculate the performance indicators: yield, purity, productivity, and concentration factor. The yield was defined as the ratio of the BSA amounts in the product and feed/Load, respectively. The product was either flow-through or lumped wash and elution fractions. The purity was the mass fraction of BSA in the product (only BSA and DNA were considered). Productivity was defined as the product mass per unit membrane volume and unit cycle time (loading, washing, elution/desorption). The concentration factor was the ratio of the BSA concentrations in the product and feed/Load, respectively. Membrane reuse was investigated under the same conditions as described above for flow-through mode. Each cycle consisted of washing with water, equilibration, loading, washing with loading buffer, desorption, and membrane regeneration with NaOH.

## 3. Results and Discussion

Interaction of mixed-mode ligands with adsorbed molecules is often complex. Ion-exchange and hydrophobic interactions are typically combined to achieve good selectivity and capacity. Therefore, a comprehensive experimental approach is required to understand their process performance and provide an optimal design of chromatographic separation processes. An essential aspect of this approach is the investigation of the effect of experimental conditions on the equilibrium and kinetic properties of multimodal adsorbents. In this work, the influence of pH, buffer ionic strength, and salt type on the adsorption of BSA and salmon DNA on a Sartobind STIC membrane was investigated. BSA was considered the model target protein, and DNA, as a larger molecule, a model impurity.

### 3.1. Membrane Pore Structure Characterization

Sartobind STIC membrane has a double-porous structure [2]. The transport pores of Sartobind support membranes have a radius of about 5 μm [17]. Secondary pores are formed directly in the regenerated cellulose matrix by the action of a porogen [2]. The total specific pore volume and porosity of Sartobind STIC membranes obtained by the liquid impregnation method were 2.8 mL/g and 0.8 mL/g, respectively. Their specific volume, determined by the batch size-exclusion method, was 2.0 mL/g. The specific volume of secondary pores, calculated as the difference between the mentioned specific pore volumes, was 0.8 mL/g.

**Figure 2:**
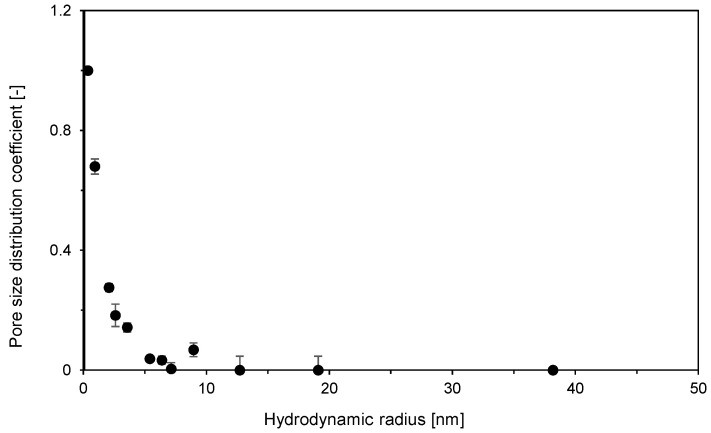
Secondary pore-size distribution coefficient vs. solute hydrodynamic radius for Sartobind STIC membranes obtained by inverse size-exclusion chromatography. The experiments were performed in triplicate, error bars represent standard deviations.

Investigation of the secondary pore structure was based on the inverse size-exclusion experiments for a set of molecular probes with the molecular weight from 180 Da (glucose) to 2000 kDa dextran. The obtained dependence of pore size distribution coefficient on the probe hydrodynamic radius is presented in Figure 2. This figure shows that dextran molecules with a molecular weight above 40 kDa were not able to access the secondary pores. The secondary pores have a radius of up to about 5 nm. The molecular weights of BSA and salmon DNA are 66 kDa [30] and 0.66 kDa per DNA base pair [31], respectively. Their molecular weights are thus higher than those of the smallest dextran excluded from the secondary pores. However, the three-dimensional structures of proteins are much more compact than those of random coils of linear dextran polymers. The hydrodynamic radius of BSA is therefore only 3.4 nm [32] and BSA can thus diffuse through the secondary pores. The DNA preparation used in this work was a polydisperse mixture of fragments ranging from 10 to 330 base pairs. This means that the linear DNA chain lengthrangess from 3.3 nm to 110 nm. Considering the DNA chain radius of 1 nm, it is evident that many of these fragments can penetrate through secondary pores relatively easily.

**Figure 3:**
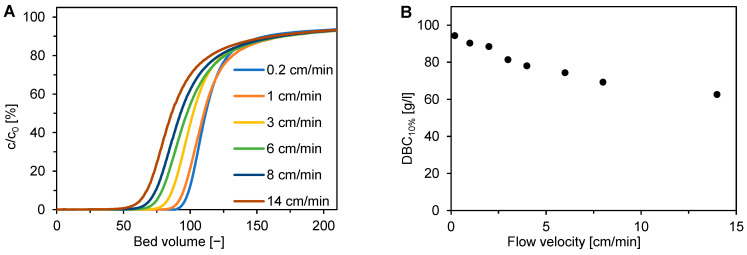
Effect of linear flow velocity on the adsorption of BSA on Sartobind STIC membranes in laboratory membrane modules. (**A**) Breakthrough curves for BSA adsorption. (**B**) Dynamic binding capacity at 10% breakthrough.

In general, membrane structure significantly influences the membrane transport properties, which are typically represented by the dynamic binding capacity (DBC). DBC is primarily a function of flow velocity. The advantage of adsorption membranes compared to particle adsorbent beds is that convective transport is dominant and DBC is much less dependent on the flow velocity. In fact, several papers presented essentially constant DBC for several proteins, including BSA, but at relatively low flow velocities, typically lower than 1 cm/min [7,12,33] and maximally at 5 cm/min [34]. In this work, we examined the effect of linear flow velocity on the DBC of BSA in a broad range from 0.2 cm/min to 14 cm/min. Figure 3 shows that DBC decreased slightly with linear flow velocity. The presented results are compatible with the previous observations in that the effect of linear flow velocity on DBC was very low at flow velocities lower than 1 cm/min. The DBC at the highest flow velocity of 14 cm/min was, however, about 30% lower due to the mass transfer resistance in secondary pores. A hypothesis on the effect of potential membrane compression at higher flow rates was excluded since the dependence between the pressure drop and flow velocity was linear.

### 3.2. Effect of Buffer Type on Single Component Adsorption

**Figure 4:**
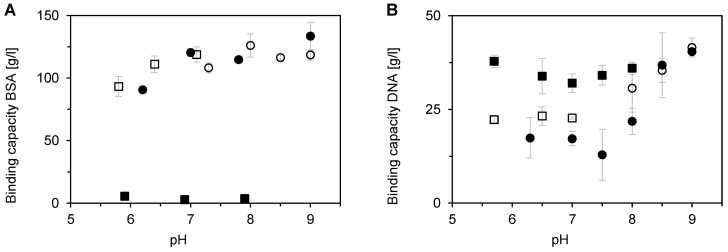
Influence of pH on single-component adsorption of BSA (**A**) and DNA (**B**) on Sartobind STIC using a micromembrane module with centrifuge-forced flow. Symbols represent effect of: (□) bis-tris buffer, (●) bis-tris propane buffer, (○) tris buffer, and (■) phosphate buffer. Error bars represent standard deviations.

The single-component binding capacity of BSA and DNA was investigated as a function of pH and buffer type. The pH range of individual buffers was chosen with respect to their buffering capacity. Figure 4 shows the binding capacities of BSA and DNA as a function of pH in the range of 5.5–9. BSA binding capacity in the phosphate buffer was very close to zero throughout the whole pH range, whereas it has rather high values (around 120–130 mg/mL at pH 7–9) in the tris, bis-tris, and bis-tris propane buffers (Figure 4A). In the latter case, the BSA binding capacity increased with pH, similarly as for BSA adsorption on the strong anion-exchange membrane Sartobind Q [2].

It has to be emphasized that the phosphate buffer had no detrimental effect on the BSA adsorption on Sartobind Q [12]. Negligible binding of BSA in the presence of polyvalent anions may be a general feature of multimodal adsorbents based on primary amine ligands. Phosphate ions can interact with hydrogen atoms and amine groups in a polymeric ligand chain, which shields the ligand binding sites from protein molecules. This behavior was also observed during the clearance of a minute virus in mice on a membrane adsorbent containing primary amine-based ligands [18].

Figure 4B shows that the effect of pH and buffer type on the DNA binding capacity for Sartobind STIC was very different from that observed in the case of BSA. The DNA binding capacity for the phosphate buffer was even higher than for other buffer types. The positive influence was more pronounced at an acidic pH. Good binding of DNA from a phosphate buffer on a multimodal membrane adsorbent was also found by Weaver et al. [35]. The pH dependence had a clear minimum at pH 7–7.5 for each buffer. The minimum adsorption capacity was only about 10 mg/mL, whereas the maximal value obtained at pH 9 was about 40 mg/mL. The smaller specific adsorbed mass of DNA compared to that of BSA can be caused by the limited accessibility of secondary pores.

### 3.3. Effect of Salt Type and Ionic Strength on Single Component Adsorption

**Figure 5:**
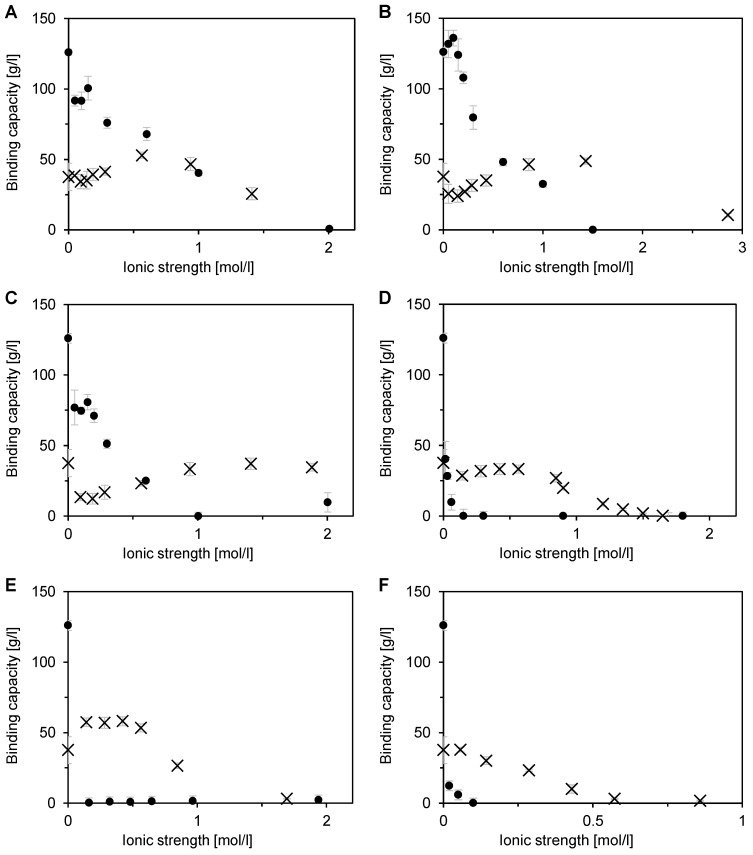
Influence of salt type and ionic strength on single-component adsorption of BSA (●) and DNA (🞨) on Sartobind STIC using a micromembrane module with centrifuge-forced flow at a pH of 8. (**A**) NaSCN, (**B**) NaCl, (**C**) NH_4_Cl, (**D**) (NH_4_)_2_SO_4_, (**E**) Na_2_SO_4_, and (**F**) NaH_2_PO_4_/Na_2_HPO_4_. Error bars represent standard deviations.

**Figure 6:**
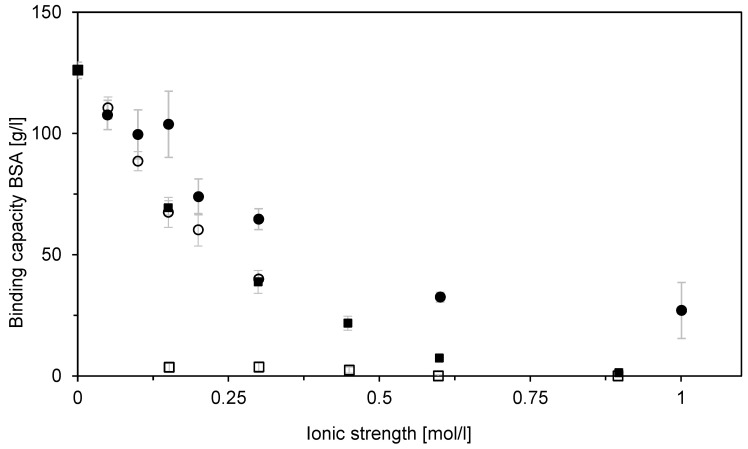
Influence of salt type and ionic strength on single-component adsorption of BSA on Sartobind STIC using a micromembrane module with centrifuge-forced flow at a pH of 8. Symbols represent GuHCl (●), MgCl_2_ (■), NaF (○), and K_2_HPO_4_ (□). Error bars represent standard deviations.

The effect of ionic strength and salt type on single-component binding capacity was investigated at a pH of 8. The selected salt ions span throughout the whole Hofmeister series, so compounds with both chaotropic and kosmotropic effects were investigated. The influence of salt type on the single-component adsorption of BSA and DNA is shown in Figure 5 and Figure 6. The affinity of BSA for the membrane adsorbent surface was greatly influenced by the ion position in the Hofmeister series. This impact was much larger than that observed for BSA adsorption on conventional IEX resins [36]. The highest level of salt tolerance was exhibited by the monovalent chaotropic salt NaSCN (Figure 5A), followed by NaCl (Figure 5B), and NH_4_Cl (Figure 5C). A pronounced capacity decrease occurred only at the ionic strength values above 200 mmol/L. The threshold concentration with zero binding capacity was between 1–2 mol/L.

A very interesting outcome was obtained for strong kosmotropic salts with polyvalent anions: (NH_4_)_2_SO_4,_ Na_2_SO_4_, NaH_2_PO_4_/Na_2_HPO_4_ (Figure 5D–F), and K_2_HPO_4_ (Figure 6). They acted as a typical displacer, so the binding capacity was already negligible at the ionic strength of 100 mmol/L. This phenomenon is common for conventional ion exchangers [37,38]. Figure 6 shows that the other examined compounds (salts and guanidium hydrochloride) with a monovalent anion were also salt-tolerant, albeit to a lesser extent than those presented in Figure 5A–C.

The influence of salt type on the binding capacity was very different when DNA was adsorbed (Figure 5). Salt tolerance was observed in all cases, but the degree of salt tolerance and the effect of ionic strength differ significantly for individual salts. Salt tolerance was more enhanced in the presence of monovalent salts (Figure 5A–C). The binding capacity was still rather high at an ionic strength of above 1 mol/L. On the contrary, Figure 5D–F shows that, for polyvalent salts, the binding capacity at an ionic strength of 1 mol/L was either low or negligible. There are, however, significant differences between the effects of individual salts in each of these two groups.

A decrease in binding capacity in the ionic strength range of about 0–200 mol/L was observed for all monovalent salts. This binding capacity drop was however very different for individual salts. It was only about 10% of the value in salt-free solution for NaSCN (Figure 5A). In the case of NaCl, the binding capacity decreased by one third (Figure 5B). The greatest decrease, from 37.6 g/L to about 12 g/L, was observed for NH_4_Cl (Figure 5C). Figure 5A–C displays a sharp increase in binding capacity from the mentioned minima. The relative increase is again highest in the case of NH_4_Cl (Figure 5C). At 1.4 mol/L, the binding capacity recovered to that of the salt-free solution. The binding capacity increased to a maximum of about 50 g/L in the presence of the other two monovalent salts (Figure 5A,B). This maximum was achieved at about 0.6 mol/L (Figure 5A) and 1.5 mol/L (Figure 5B). A gradual decrease of the binding capacity was observed at higher ionic strengths.

A somewhat simpler pattern of ionic strength influence was observed for polyvalent salts (Figure 5D–F). A slight decrease in the binding capacity at low salt concentrations was found only for (NH_4_)_2_SO_4_ (Figure 5D). A clear increase in the binding capacity was exhibited only in the case of Na_2_SO_4_ (Figure 5E). In both cases, the significant gradual decrease of the binding capacity started at an ionic strength of 0.5 mol/L. An even faster decrease of the binding potential occurred in the phosphate buffer, where, at 0.5 mol/L, DNA practically did not bind to the membrane (Figure 5F). Polyvalent salts are thus less favorable than the monovalent ones, also for DNA binding, albeit to a lesser extent than for proteins. These results imply almost fully selective binding of DNA in the presence of polyvalent ions, which could be exploited in the flow-through polishing steps. On the other hand, monovalent salts seem to be more convenient for other separation modes.

**Figure 7:**
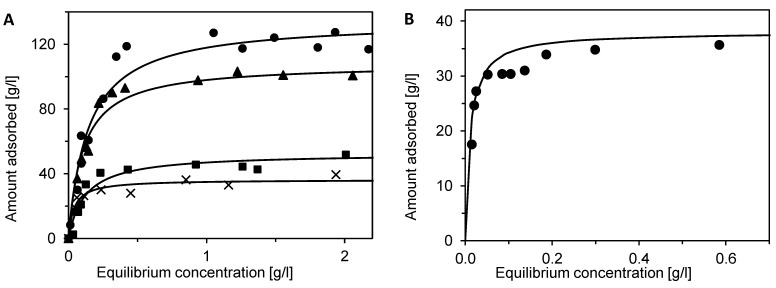
Adsorption isotherms of BSA (**A**) and DNA (**B**) on Sartobind STIC at different NaCl concentrations: (●) 0 M, (▲) 0.15 M (■) 0.4 M, and (🞨) 1 M. Lines represent Langmuir adsorption isotherm approximations.

Both BSA and DNA have high affinity toward Sartobind STIC, as follows from the illustrative adsorption isotherms shown in Figure 7. The maximum saturation capacities q_max_ (Figure 7 and Table 1) were often achieved at equilibrium concentrations of about 0.1–0.2 g/L. These BSA and DNA concentrations were essentially independent of salt concentration. This was confirmed by the affinity constant values of the Langmuir isotherm. Table 1 shows that, for BSA, they were about 10 l/g.

### 3.4. Effect of Eluent Composition on BSA and DNA Recovery

The choice of eluent has a significant effect on separation in the bind-and-elute mode as well as on the recovery of bound molecules. During the elution phase, unfolding of the protein can occur, which can lead to the formation of aggregates and difficult desorption. [39,40,41]. Roberts and Carta observed that the rate of aggregate formation and the recovery of adsorbed proteins on multimodal adsorbents depended significantly on the incubation time [42,43]. Therefore, the influence of eluent composition and the effect of incubation on the desorption of BSA or DNA were investigated in this work. Several eluents were chosen that exhibited either negligible or very low binding capacities in single-component adsorption experiments (Figure 4 and Figure 5). The goal was to find suitable conditions for the elution phase. The effect of incubation of adsorbed components on their recovery was investigated so that the membrane with adsorbed BSA/DNA was washed with the equilibration buffer and allowed to incubate for 12 h prior to desorption.

**Figure 8:**
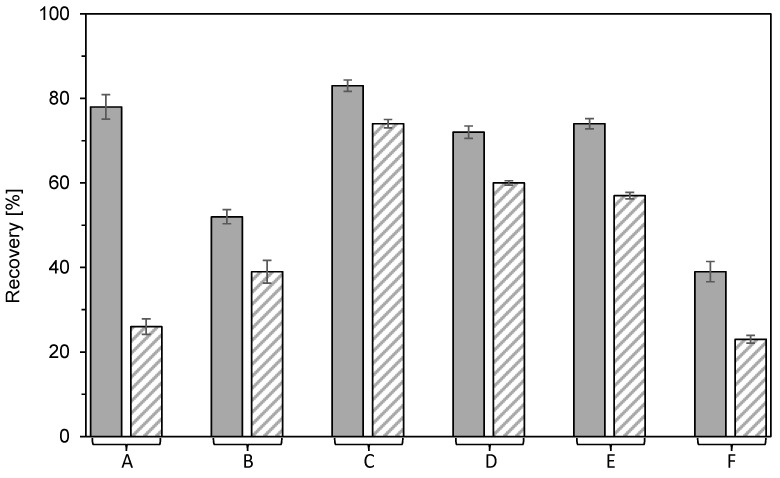
Effect of eluent composition (**A**) 2 M NaCl in tris pH 8, (**B**) phosphate buffer pH 5.8, (**C**) 2 M NaCl in phosphate buffer pH 5.8, (**D**) citrate-phosphate buffer pH 3.6, and (**E**) 0.5 M Na_2_SO_4_ in tris pH 8 on BSA recovery, and (**F**) 2 M NaCl in tris pH 8 on DNA recovery. Grey bars represent results for immediate elution after adsorption and washing steps (no incubation). Bars with stripes represent the recovery of membranes that were incubated for 12 h after the adsorption and washing steps. Error bars represent standard deviations.

The influence of eluent composition on the recovery of BSA is shown in Figure 8. Recovery of BSA using 2M NaCl in tris buffer as eluent was approximately 78% for a non-incubated membrane but only 26% for the incubated one (bars A in Figure 8). This difference may be due to the increased hydrophobic interactions of the unfolded protein with the adsorbent. Only 52% and 39% of adsorbed BSA (bars B), respectively, were recovered using the pure phosphate buffer with pH 5.8. The low recovery value for the non-incubated membrane is partly surprising since a zero binding capacity was observed for the pure phosphate buffer in the single-component adsorption experiments. The low recovery also implies problems with the reversibility of BSA adsorption.

The addition of 2 M NaCl to the phosphate buffer significantly improved the desorption of bound BSA even for the incubated membrane (bars C). The polyvalent ions of the phosphate buffer disrupted the hydrophobic interactions of BSA with the multimodal adsorbent, and the high concentration of NaCl ensured the displacement of adsorbed BSA. When a citrate phosphate buffer with a pH of 3.6 was used, recovery rates of 72% and 60%, respectively, were achieved (bars D). Evidently, the low pH had a positive effect on BSA desorption, but its applicability is questionable since it can contribute to the formation of aggregates. A similar positive effect had an addition of a salt kosmotropic salt with polyvalent anions. The recoveries of 74% and 60%, respectively, were achieved when 0.5 M Na_2_SO_4_, in a tris buffer with pH 8 was used as eluent.

The same approach was chosen to investigate DNA desorption (Figure 8, bars F). In the case of DNA desorption, a maximum recovery of 40% (23% for the incubated membrane) was achieved using 2 M NaCl in the tris buffer. Such low recovery values indicates that DNA is very strongly bound to the multimodal adsorbent and its desorption is difficult. This troublesome desorption of DNA limits the potential of Sartobind STIC for the purification of DNA. It should be mentioned here that some multimodal adsorbents were used for the purification of DNA fragments [44], DNA plasmids [45], and minicircles [46] intended for gene therapy or DNA vaccination, but a thorough optimization of adsorption and desorption conditions had to be made [47].

### 3.5. Effect of Salt Type and Concentration on Two-Component Adsorption

Although single-component adsorption experiments indicate the effect of the mobile phase composition on the separability of model molecules, adsorption of a multicomponent mixture can provide a different picture due to the competition effect. As it was shown above, competitive binding of proteins and nucleic acids is more significant for monovalent salt solutions, whereas separation is simpler for polyvalent salts. In order to investigate these hypotheses, model salts were chosen for two-component adsorption experiments. These were the monovalent salts NaCl and NH_4_Cl, and the polyvalent salt Na_2_SO_4_.

**Figure 9:**
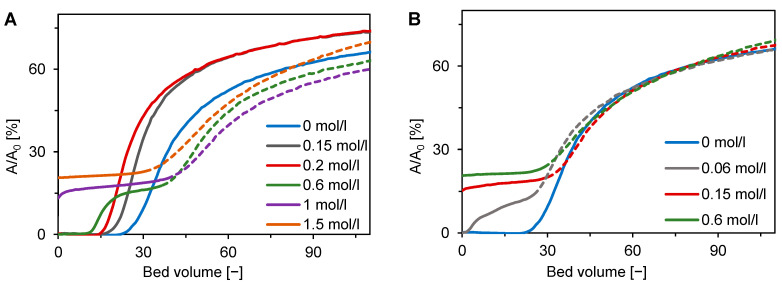
Influence of salt type and ionic strength on two-component adsorption of BSA and DNA on Sartobind STIC using a micromembrane module with pump-forced flow at pH 8. (**A**) NaCl and (**B**) Na_2_SO_4_. Breakthrough curves for different ionic strengths, expressed via relative absorbance, are distinguished by different colors. The solid and dashed lines correspond to the first and second adsorption fronts in the breakthrough, respectively.

In the two-component micromembrane experiments, outlet stream UV absorbance and BSA and DNA concentrations in the pooled outlet fractions were measured. Figure 9A,B present the influence of NaCl and Na_2_SO_4_ concentration on the BSA/DNA breakthrough behavior (only selected curves are shown). The measured absorbance is a superposed signal, and thus a single front was observed at some concentrations. At low salt concentrations, the breakthrough curves do not show a clear separation of BSA and DNA. On the contrary, this is visible at higher salt concentrations, where two distinct fronts occur. Following the results of single-component adsorption, the earlier breakthrough front can be associated with BSA and the later one with DNA.

The latest initial breakthrough occurred at a salt concentration of 0 mol/L (Figure 9A,B). This observation corresponds well with both components having a high binding capacity in salt-free conditions (Figure 5). The initial breakthrough time decreased with the salt concentration for both single- and double-front breakthrough curves. The single-front curves were observed for NaCl concentrations up to 0.2 mol/L (Figure 9A). The decrease of the initial breakthrough time can be explained by significant decrease of the DNA binding capacity at low NaCl concentrations (Figure 5B). Figure 5B can also explain the existence of two fronts formed above the concentration of 0.2 mol/L when the DNA binding capacity increases whereas that of BSA decreases.

Figure 9B shows that, in the case of Na_2_SO_4_, two-front breakthrough curves appeared already at an ionic strength as low as 0.02 mol/L. This result is also compatible with the single-component equilibrium data presented in Figure 5E when the BSA binding capacity decreased very quickly to its zero value, which then results in an instantaneous breakthrough of BSA as observed for 1 mol/L NaCl (Figure 9A) and 0.05 mol/L Na_2_SO_4_ (Figure 9B).

The BSA and DNA content in pooled outlet fractions allowed determining their bed-adsorbed amounts. In the absence of salts, the bed-adsorbed amounts of BSA and DNA were 43 g/L and 17.5 g/L, respectively. The total adsorbed amount of 60.5 g/L is between the single-component binding capacity values of DNA and BSA, which were 38 g/L and 126 g/L, respectively. This result implies that the adsorbent had a higher affinity for DNA in salt-free conditions as well. As expected from the single-component adsorption data, the DNA bed-adsorbed amounts during competitive binding were higher in the presence of salts. The maximum values were 23.5 g/L for NaCl, 26.7 g/L for NH_4_Cl, and 25 g/L for Na_2_SO_4_.

**Figure 10:**
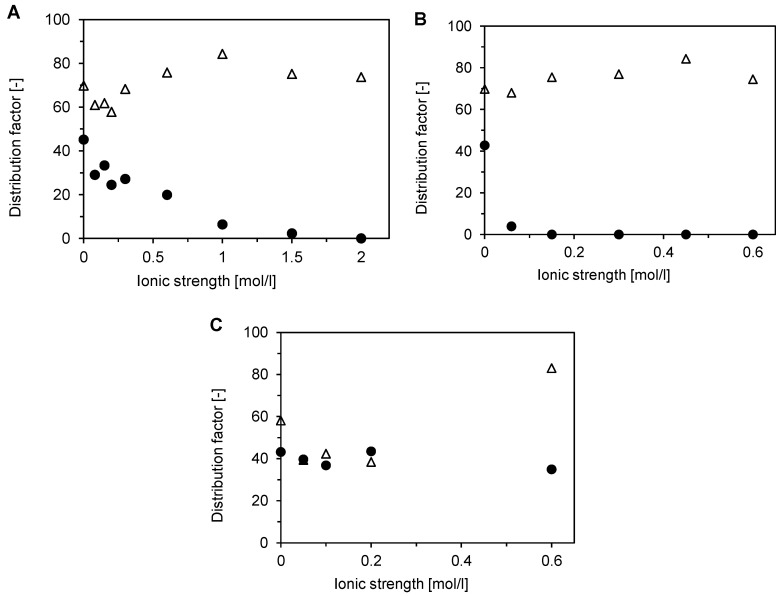
Influence of salt type and ionic strength on two-component adsorption of BSA (●) and DNA (△) on Sartobind STIC using a micromembrane module with pump-forced flow at pH 8. (**A**) NaCl, (**B**) Na_2_SO4, and (**C**) NH_4_Cl.

The measured bed-adsorbed amounts were used to calculate distribution factors (Equation (5)), which are presented for all three examined salts as a function of ionic strength in Figure 10. The dependences shown in Figure 10 are compatible with those obtained for single-component adsorption (Figure 5), but there are some differences in the trends. In the case of NaCl, the DNA distribution factor had a much less pronounced minimum at low ionic strength and a maximum at about 1–1.5 mol/L (Figure 10A). Minimal differences in the shapes were observed for Na_2_SO_4_ (Figure 10B). The largest differences were observed for NH_4_Cl (Figure 10C). The distribution factor of BSA was essentially constant in contrast to the sharp decline of BSA single-component binding capacity (Figure 5C). Similarly, the decrease of the DNA distribution factor at low ionic strengths (Figure 10C) was much lower than that of single-component binding capacity (Figure 5C).

**Figure 11:**
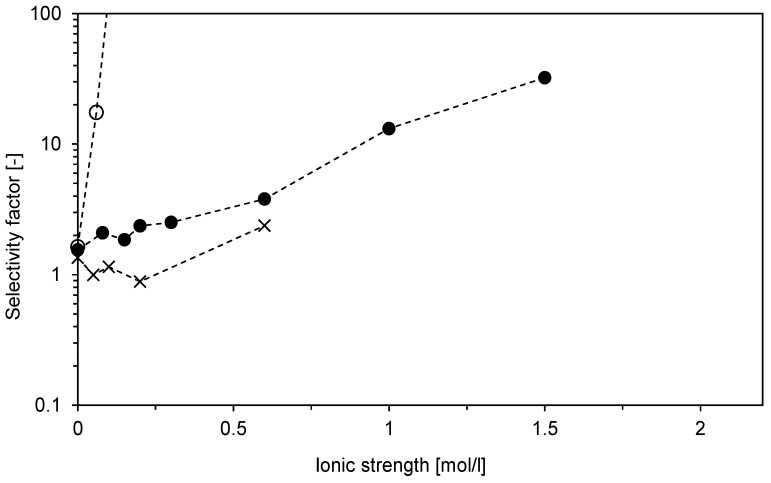
Influence of salt type and ionic strength on two-component adsorption of BSA and DNA on Sartobind STIC using a micromembrane module with pump-forced flow at a pH of 8. The selectivity factor represents the ratio of distribution factors plotted in Figure 7. NaCl (●), NH_4_Cl (🞨), and Na_2_SO_4_ (○). Connecting lines are included only for better visualization of trends.

The results presented in Figure 10 demonstrate the favorable binding of DNA. The selectivity factor, defined by Equation (6), quantifies the potential of the Sartobind STIC adsorbent to separate the model target and impurity molecules. Figure 11 shows that the best separation selectivity can be achieved for Na_2_SO_4_ when the selectivity factor tends to infinite values at low salt concentrations. On the contrary, NH_4_Cl allows very limited selectivity at low salt concentrations. The selectivity factor for NaCl increases gradually from values of about 2–3 at concentrations below 300 mM to values above 10 at concentrations of 1 mol/L and higher. This good selectivity at high salt concentrations allows using this membrane in a downstream processing train directly, without any feed dilution or diafiltration. Moreover, the high selectivity factor makes it suitable for the flow-through mode.

### 3.6. Bind-and-Elute and Flow-Through Mode Separation

**Figure 12:**
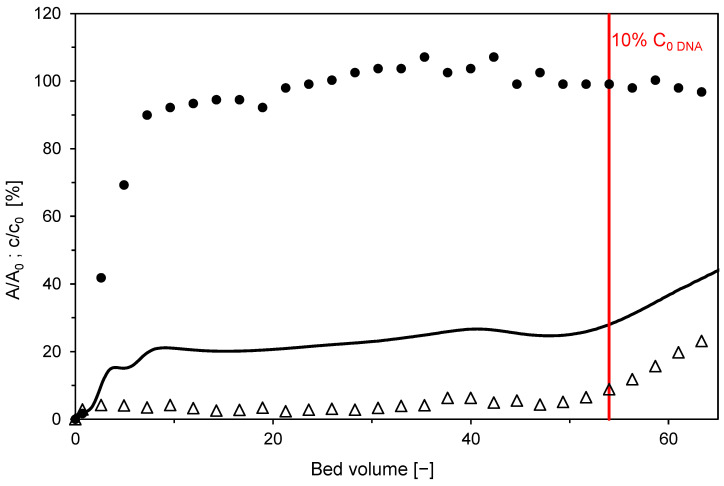
Two-component adsorption of BSA (●) and DNA (△) on Sartobind STIC from a feed containing 0.15 mol/L of Na_2_SO_4_ using the laboratory membrane module in flow-through mode. Symbols represent the relative concentrations of BSA and DNA in the collected fractions. The solid black line is the relative outlet stream absorbance which is a superposition of BSA and DNA outlet concentrations. Red line indicates the moment of 10% breakthrough of DNA.

Scale-up of the results of micromembrane experiments, including the verification of optimal conditions, was carried out in a laboratory membrane module for both flow-through and bind-and-elute modes. The feed in both modes contained the same amounts of BSA and DNA as in previous experiments: 1 g/L and 0.3 g/L, respectively. The feed in the flow-through mode furthermore contained 0.15 mol/L of Na_2_SO_4_, which corresponded to an ionic strength of 0.45 mol/L. Figure 12 shows the results of the negative flow-through mode experiment. Excellent selectivity was achieved when the BSA breakthrough was instantaneous, and 10% DNA breakthrough occurred at 54 BV. This result is compatible with the equilibrium data presented in Figure 10C and Figure 11. The analysis of pooled outlet fractions provided BSA at 99% purity (96.8% DNA reduction) and with a yield of 97% (Table 2). The BSA and DNA dynamic binding capacities at the 10% breakthrough were 1.55 g/L and 15.4 g/L, respectively.

**Figure 13:**
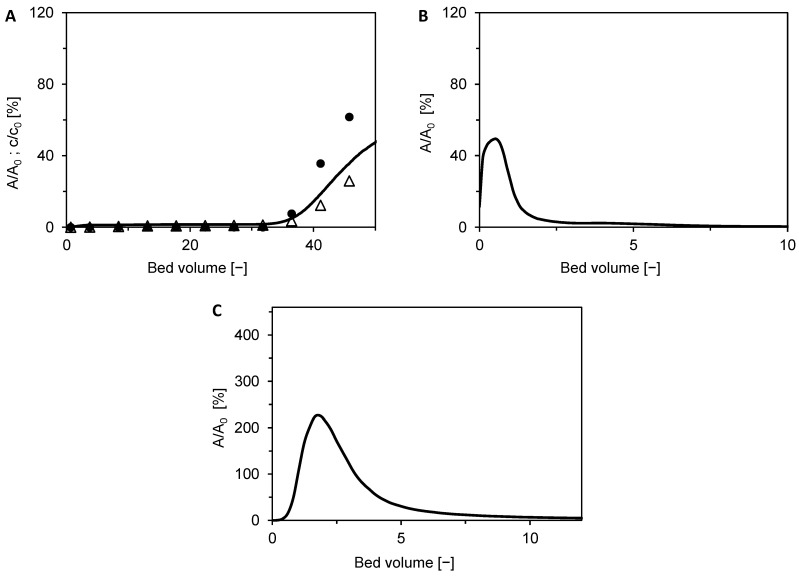
Two-component adsorption of BSA (●) and DNA (△) on Sartobind STIC using the laboratory membrane module in bind-elute mode with a loading of 48 BV. (**A**) Adsorption phase using salt-free feed. (**B**) Washing phase. (**C**) Elution phase using 0.15 mol/L Na_2_SO_4_ as eluent. Symbols represent the relative concentrations of BSA and DNA in the collected fractions. The solid black line is the relative outlet stream absorbance, which is a superposition of BSA and DNA outlet concentrations. Due to the negligible content of DNA in the outlet stream of the washing and elution phases, respectively, the chromatograms in Figure 13B,C essentially represent BSA content in the outlet stream.

Bind-and-elute mode experiments were performed for two different loadings, 48 BV and 18 BV, respectively. Figure 13 shows chromatograms for the loading of 48 BV. Both BSA and DNA bound very well during the adsorption phase in salt-free conditions (Figure 13A). An initial breakthrough of both components occurred approximately at 35 BV, and the dynamic binding capacities of BSA and DNA at this moment were 37.8 g/L and 11.4 g/L, respectively. As follows from the breakthrough curve of BSA, the membrane was nearly saturated with this protein at the end of the loading phase. Figure 13B displays a small peak of BSA in the washing phase, which probably corresponds to unbound BSA in the membrane pores and module dead volume.

BSA was selectively desorbed with Na_2_SO_4_ in the elution phase, as it follows from the chromatogram in Figure 13C and the determined BSA purity of 100% (99.5% of DNA removed) in Table 2. The yield was however, lower than in the flow-through mode—only 65%. A somewhat higher yield of 80% was achieved for a loading of 18 BV (Table 2), owing to no loss of BSA either in the adsorption phase or during the washing step (data not shown). Nonetheless, incomplete recovery of BSA is compatible with the results of our previous study on the rhEPO separation using the Sartobind STIC membrane [48]. Poor reversibility of protein binding is however a common problem of multimodal adsorbents [26,33,49,50].

A positive aspect of the bind-elute mode is that BSA was concentrated by a factor of about 2.5 (Table 2). However, the negative flow-through mode can provide not only a significantly higher BSA yield but also about twice as much productivity. High yield and productivity result from the negligible BSA binding in the presence of Na_2_SO_4_ (Figure 5E and Figure 10B). This observation can be generalized so that the flow-through mode can be advantageous for protein product downstream processing if a highly selective multimodal adsorbent is used to bind impurities. A particular advantage of multimodal membranes in this regard is that they can cope with much larger impurity biomolecules or viruses than particle adsorbents.

### 3.7. Membrane Reuse

Multimodal beads are commonly used for repeated use. On the contrary, membrane adsorbents are designed for single use, which is associated with higher adsorbent-associated costs in the production processes. Their application is economically attractive if they can be used in flow-through mode, ideally in the case when short manufacturing campaigns are required [51]. Osuofa et al. demonstrated that multimodal membrane adsorbents could be used repeatedly in 100 cycles when the desorption and regeneration steps in the bind-and-elute mode were optimized [27].

**Figure 14:**
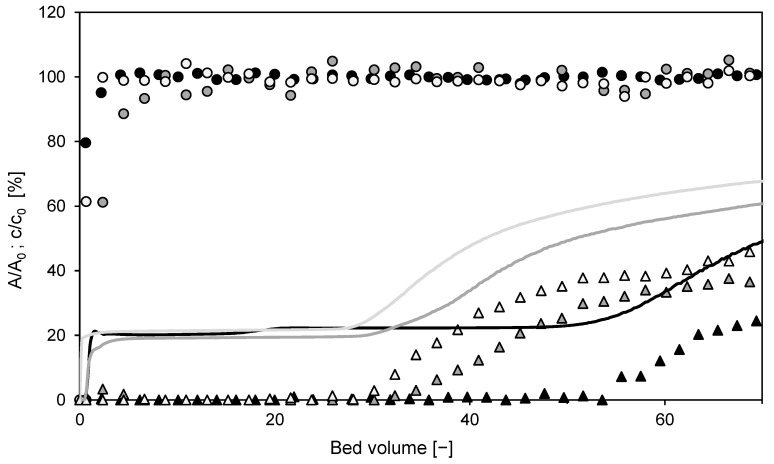
Overlay of chromatograms from repeated cycles using the laboratory membrane module in flow-through mode. The solid line is the relative outlet stream absorbance, which is a superposition of BSA and DNA outlet concentrations. Symbols represent the relative concentration of BSA (●) and DNA (△) in collected fractions. The color of lines and symbols distinguishes individual cycles: black–1st cycle, gray–2nd cycle, and light gray-3rd cycle.

As was shown above, satisfactory separation of the model protein BSA from the DNA impurity was achieved in the negative flow-through mode using 0.15 M Na_2_SO_4_. The product was in the flow-through, and the impurities were adsorbed on the membrane. To reuse the membrane, the DNA was desorbed with 2 M NaCl and regenerated with 1 M NaOH. The membrane that had been regenerated in this way was ready to be used in the next cycle. In total, three cycles were applied. Chromatograms of the individual cycles are shown in Figure 14. As can be observed, the breakthrough of DNA occurred earlier in the second and third cycles. DNA dynamic binding capacity (at 10% breakthrough) thus decreased with the number of cycles. It was 16.1 g/L for the first cycle, 10.6 g/L for the second cycle, and 8.9 g/L for the third one. It is not clear whether this decrease was caused by ligand degradation, blocking of functional sites by aggregates, degradation of the support, or non-specific adsorption of proteins in intraparticle pores. To clarify the exact mechanism behind this performance loss, further investigation would be needed. The earlier DNA breakthrough in the repeated cycles decreases the bed loading and adsorption processes’ productivity.

## 4. Conclusions

This work deals with adsorption properties of a multimodal salt-tolerant anion-exchange membrane Sartobind STIC based on a regenerated cellulose support with a poly(allylamine) ligand. This adsorbent possesses a bimodal pore structure with micrometer-size transport pores and secondary pores in the cellulose matrix. The secondary pore-size distribution was examined using inverse size-exclusion chromatography, providing a radius of up to about 5 nm. They were thus accessible for BSA and salmon DNA fragments, which were chosen as the model protein product of interest and the key impurity, respectively.

Several single- and two-component adsorption experiments were carried out using a micromembrane chromatography module based on a 96-well plate. It was found that the buffer type has a significant effect on the adsorption equilibrium. The BSA binding capacity in the phosphate buffer was negligible at any pH, whereas it was rather high for other buffer types. The negative effect of the polyvalent phosphate ions was explained by their interaction with hydrogen atoms in the polymeric ligand chain amine groups. On the contrary, DNA binding capacity was the highest in the phosphate buffer. These results indicate that the interplay between ion-exchange and hydrophobic interaction mechanisms of this multimodal adsorbent is rather complex.

For a more thorough analysis of Sartobind STIC equilibrium adsorption properties, single-component experiments for a wide range of anions and cations from the Hofmeister series were designed. These included compounds with both chaotropic and kosmotropic effects as well as those with monovalent or polyvalent ions. It was found that BSA adsorption is significantly influenced by the ion position in the Hofmeister series. Nonetheless, salt tolerance was observed for both chaotropic and kosmotropic monovalent salts. On the contrary, strong kosmotropic salts with polyvalent anions acted as BSA displacers at very low values of ionic strength.

The effect of the Hofmeister series ions on single-component DNA adsorption were very different. Similarly, as mentioned above for the effect of the buffer type, polyvalent ions had no negative effect on the DNA binding capacity. Salt-tolerance was thus observed for all examined salts but was more enhanced in the presence of monovalent salts. The dependence of ionic strength on the binding capacity was however not monotonous as in case of BSA. At low ionic strength, minimum binding capacity was observed in the range of 0–200 mol/L. This decrease was more pronounced for the monovalent salts than for the polyvalent ones.

At moderate values of ionic strength, the DNA binding capacity strongly increased up to a maximum. In some cases, the maximum binding capacity was even higher than the value for salt-free conditions. Again, the degree of the increase was much higher in the case of monovalent salts. Although the addition of polyvalent salts resulted in a lower DNA binding capacity, the results obtained for BSA single-component adsorption implied that DNA can be bound almost selectively and separated in the flow-through mode. It was found that incubation of adsorbed membrane in a buffer solution promoted BSA or DNA unfolding and stronger hydrophobic interactions. Such bound molecules could not be desorbed effectively. Desorption conditions were optimized and 80% recovery of BSA was achieved using 2 M NaCl or 0.5 M Na_2_SO_4_. Unfortunately, DNA could not be fully desorbed from the membrane under any conditions, which resulted in a reduced dynamic binding capacity with multiple uses of the membrane.

Competitive BSA/DNA adsorption was investigated for three selected salts in subsequent micromembrane module flow-through experiments. The effect of ionic strength was also investigated in this series of experiments. The obtained breakthrough curves showed a clear separation line between BSA and DNA when BSA breakthrough was almost instantaneous at an ionic strength exceeding a certain value. The best separation performance was achieved for Na_2_SO_4_ due to its enormously high selectivity factor at salt concentrations as low as 0.05 M.

The conclusions of the screening and optimization experiments were verified at a larger scale using a laboratory membrane module thattt allowed BSA and DNA determination in pooled fractions in both negative flow-through mode and bind-elute mode. The mass transfer effects on the separation performance of the membrane module were rather low, as indicated by a small decrease in the dynamic binding capacity when the flow velocity was varied by a factor of 70. The operation in the negative flow-through mode was clearly more advantageous when BSA was obtained at 99% purity and with a 97% yield. The productivity was also about twice as high as in the bind-elute mode experiments with different loadings. The latter mode also provided essentially pure BSA, but the yield did not exceed 80%. The incomplete BSA recovery can be explained by the poor reversibility of its binding to the multimodal adsorbent.

## Figures and Tables

**Table 1:** Parameters of the Langmuir isotherm for the equilibrium data presented in Figure 7.

c_NaCl_ [M]	q_max_ [g/L]	K [l/g]
0	134	7
0.15	108	10
0.4	53	8
1	36	25
0 *	38	90

* Parameters of the Langmuir adsorption isotherm for DNA.

**Table 2:** Performance indicators for BSA purification on Sartobind STIC using the laboratory membrane module in the negative flow-through mode and bind-and-elute mode, respectively.

Operation Mode	Yield [%]	Purity [%]	Productivity [mg/mL/min]	Concentration Factor
Flow-through *	97	99.0	11	0.96
Bind-elute, loading 48 BV	72	99.8	4.52	2.7
Bind-elute, loading 18 BV	80	99.6	4.8	2.2

* Indicators were calculated for 10% breakthrough of DNA which occurs approximately at 54 BV.

## Data Availability

Data are contained within the article.
